# The implementation of a community-based aerobic walking program for mild to moderate knee osteoarthritis (OA): a knowledge translation (KT) randomized controlled trial (RCT): Part I: The Uptake of the Ottawa Panel clinical practice guidelines (CPGs)

**DOI:** 10.1186/1471-2458-12-871

**Published:** 2012-10-13

**Authors:** Lucie Brosseau, George A Wells, Glen P Kenny, Robert Reid, Andreas Maetzel, Peter Tugwell, Maria Huijbregts, Carolyn McCullough, Gino De Angelis, Lily Chen

**Affiliations:** 1 University Research Chair, School of Rehabilitation Sciences, University of Ottawa, Ottawa, Canada; 2 Department of Epidemiology and Community Medicine, University of Ottawa, Ottawa, Canada; 3 School of Human Kinetics, Faculty of Health Sciences, University of Ottawa, Ottawa, ON, Canada; 4 University of Ottawa Heart Institute, Ottawa, ON, Canada; 5 University of Toronto, Toronto, Canada; 6 Centre for Global Health, Institute of Population Health, University of Ottawa, Ottawa, Canada; 7 Baycrest Centre, Toronto, Canada; 8 Inter-Action Rehabilitation Inc, Toronto, ON, Canada

**Keywords:** Osteoarthritis, Clinical trial, Walking, Adherence, Education, Behavioural intervention, Guidelines implementation, Knowledge translation

## Abstract

**Background:**

The implementation of evidence based clinical practice guidelines on self-management interventions to patients with chronic diseases is a complex process. A multifaceted strategy may offer an effective knowledge translation (KT) intervention to promote knowledge uptake and improve adherence in an effective walking program based on the Ottawa Panel Evidence Based Clinical Practice Guidelines among individuals with moderate osteoarthritis (OA).

**Methods:**

A single-blind, randomized control trial was conducted. Patients with mild to moderate (OA) of the knee (n=222) were randomized to one of three KT groups: 1) Walking and Behavioural intervention (WB) (18 males, 57 females) which included the supervised community-based aerobic walking program combined with a behavioural intervention and an educational pamphlet on the benefits of walking for OA; 2) Walking intervention (W) (24 males, 57 females) wherein participants only received the supervised community-based aerobic walking program intervention and the educational pamphlet; 3) Self-directed control (C) (32 males, 52 females) wherein participants only received the educational pamphlet. One-way analyses of variance were used to test for differences in quality of life, adherence, confidence, and clinical outcomes among the study groups at each 3 month assessment during the 12-month intervention period and 6-month follow-up period.

**Results:**

Short-term program adherence was greater in WB compared to C (p<0.012) after 3 months. No statistical significance (p> 0.05) was observed for long-term adherence (6 to 12 months), and total adherence between the three groups. The three knowledge translation strategies demonstrated equivalent long-term results for the implementation of a walking program for older individuals with moderate OA. Lower dropout rates as well as higher retention rates were observed for WB at 12 and 18 months.

**Conclusion:**

The additional knowledge translation behavioural component facilitated the implementation of clinical practice guidelines on walking over a short-term period. More studies are needed to improve the long-term walking adherence or longer guidelines uptake on walking among participants with OA. Particular attention should be taken into account related to patient’s characteristic and preference. OA can be managed through the implementation of a walking program based on clinical practice guidelines in existing community-based walking clubs as well as at home with the minimal support of an exercise therapist or a trained volunteer.

**Trial Registration:**

Current Controlled Trials IRSCTNO9193542

## Background

Rising healthcare costs, limited resources, and the aging population have created new and growing challenges in the management of osteoarthritis (OA). The challenges associated with the management of OA are numerous as the prevalence of the population continues to increase while healthcare resources remain limited [1-7]. It is therefore necessary to determine the most effective methods of integrating research evidence in order to optimize health outcomes.

The purpose of this randomized controlled trial (RCT) was to compare 1) improvements in quality of life (QoL) and clinical outcomes such as pain, mobility and endurance); 2) adherence rates; and 3) confidence and self-efficacy after the implementation of a 12-month supervised community-based aerobic walking program (SCAWP) based on the Ottawa Panel clinical practice guidelines (CPG) among three knowledge translation (KT) intervention arms. QoL, confidence, and self-efficacy were compared at 12- months (end of treatment) and at 18-months (6-months post-intervention). Adherence was compared during the intervention period at 3, 6, 9 and 12 months.

The first part of this manuscript introduces each KT intervention and demonstrates the impact of knowledge (CPG) uptake of each by comparing outcomes influenced by the KT intervention such as adherene and behaviour change (confidence and self-efficacy). Knowledge application of each KT intervention is explained through the use a theoretical framework: Knowledge-To-Action Cycle (KTAC) [8,9]. The second part of this manuscript focuses on outcome evaluation, a specific phase in the KTAC framework used to guide this study. Part II demonstrates the effect of each intervention involving a walking program on outcomes which were influenced by the SCAWP including QoL (primary outcome) and other clinical outcomes such as pain, mobility, and endurance. These outcomes are exhibited in part II. Therefore, both parts of this manuscript were split according to the ‘’Evaluate outcomes” phase of the KTAC framework. Part I focused on KT outcomes which measured the success of CPG uptake through participants’ adherence and behavioural change while part II focused on clinical outcomes which measured the positive effect of SCAWP due to the indirect CPG uptake/implementation/adoption by individuals with OA of the knee.

### Knowledge translation

A major issue in health research today is finding effective and efficient ways to exchange knowledge between researchers, clinicians and the general public. Potential benefits to health which could be accrued through the application of research findings are not realized due to challenges in research uptake and knowledge translation [10]. KT is defined by Estabrooks et al. [11] as *“the process by which specific research-based knowledge (science) is implemented in practice.”*

The available evidence suggests that with an effective KT strategy, uptake of evidence-based clinical practice guidelines (EBCPGs) can be effective in improving patient health outcomes [12].

### Physical activity and osteoarthritis

The promotion of physical activity (PA) is a priority for health organizations serving the general population [13,14] and is highly recommended for subgroups affected by chronic diseases [15-19]. Community-based PA combined with a behavioural modification and self-management interventions can reduce the risk of disability and negative consequences of inactivity related to OA [20,21]. The challenge is to develop PA programs that will encourage OA patients to initiate and maintain improvements in exercise behaviour over a long-term period [22]. The available evidence suggests that with an effective KT strategy, uptake of evidence-based clinical practice guidelines (CPGs) can be effective in improving patient health outcomes [12].

### Selecting an effective KT strategy

Multifaceted interventions combining more than one KT strategy have tended to be more effective than using a single KT strategy alone [23,24] and have shown to have the greatest impact on CPG adherence and PA performance [25]. Behavioural interventions have been used for other chronic conditions to improve long-term adherence and maintenance of PA regimens with varying success. The efficacy of different behavioural interventions including patient education, health counselling, goal setting, and social/peer support, delivered separately and or in combination, have been examined in the management of arthritis [26-31].

## Methods

The following methodology is in full agreement with the Consolidated Standards of Reporting Trials (CONSORT) 2010 Statement criteria for reporting RCTs [32].

### Design

This single blind RCT, funded by the Canadian institutes of Health Research, used a parallel group design (1:1:1). This community-based study was approved by the University of Ottawa Research Ethics Board and the City of Ottawa Public Health Research Ethics Board. This study implemented one of the Ottawa Panel CPG recommendations related to a supervised community-based aerobic walking programs (SCAWPs) among individuals with mild to moderate OA of the knee [33-35].

**Figure 1:**
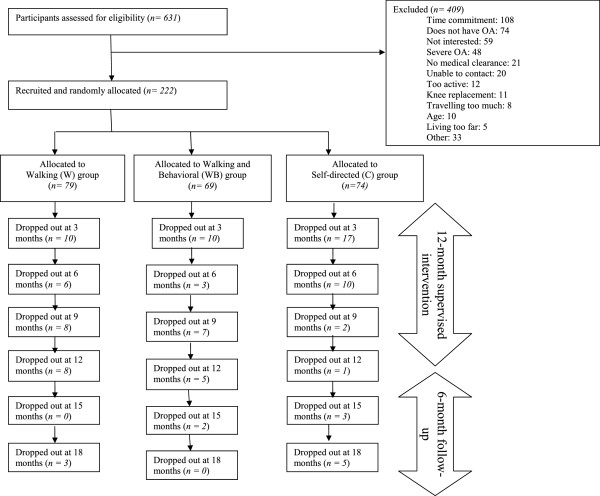
**Knowledge-To-Action Cycle.** This figure illustrates the Knowledge-To-Action Cycle Framework [8]. This figure is used with permission: Lost in knowledge translation: Time for a map. Graham ID, et al. *Journal of Continuing Education in the Health Professions, 26*(1). Copyright © 2006. The Alliance for Continuing Medical Education, The Society for Academic Continuing Medical Education, and The Association for Hospital Medical Education.

In order to facilitate an understanding of the KT process, Graham et al. developed a conceptual framework entitled “the Knowledge-To-Action Cycle” (Figure 1) which provides an approach which combines commonalities from various planned-action theories [8,9]. This framework was used to guide the development of the three KT strategies used in this RCT (Additional file 1). The framework demonstrates the dynamic process of knowledge creation and application [9]. Knowledge creation consists of three phases: knowledge inquiry, knowledge synthesis, and knowledge tools/products. The knowledge creation cycle of the framework demonstrates how knowledge is refined through each phase to provide useful information for end users. The action cycle of the framework consists of seven phases which may occur sequentially or simultaneously: 1) Identify the Knowledge-To-Action Cycle gaps; 2) Adapt Knowledge to local context; 3) Assess barriers to Knowledge Use; 4) Select, Tailor, Implement Interventions; 5) Monitor Knowledge Use; 6) Evaluate outcomes; and 7) Sustained Knowledge Use. For part I, the emphasis was placed on the “Evaluate outcomes (KT outcomes)” phase as participants’ knowledge uptake was measured as adherence to the effective walking program for OA and attitude/behaviour change (self-efficacy and confidence) after participating in the SCAWP.

The hypothesis was that the additional behavioural approach of the multifaceted KT intervention would: 1) increase short and long term adherence to the SCAWP; 2) reduce the drop-out rate; 3) influence behaviour change by improving self-efficacy, confidence, QoL and other clinical outcomes.

### Sample and recruitment

A total of 222 adults with knee OA were recruited. Participants were eligible to participate in the study if he/she 1) had a confirmed diagnosis with mild to moderate unilateral or bilateral OA according to the American College of Rheumatology clinical and radiographic/magnetic resonance imagery criteria; 2) reported pain for at least 3 months; 3) expected his/her medication to change during the study period; 4) demonstrated an ability to ambulate for a minimum of 20 minutes, at their own pace with minimal reports of pain (≤ 3 out of 10 on a visual analogue pain rating scale) [36]; 4) were able to be treated as an out-patient; and 5) were available three times a week over a period of 12 months. Potential study subjects were excluded if they had: 1) Participated in regular physical or aerobic sports ≥ 2 times per week for more than 20 minutes per session during the previous 6 months; 2) severe OA of the knee or other weight bearing joints of the lower extremity; 3) no written consent from their physician to participate in the study; 4) pain at rest or at night; 5) received rehabilitation treatment, corticosteroids injection, or any other pain-related treatment besides medication for arthritis within the last 12 months; 6) uncontrolled hypertension (Systolic blood pressure >160 mm Hg confirmed by the screening initial VO2 max test at the Ottawa Heart Institute) 7) other illnesses, such as rheumatoid arthritis (judged by the patient or study physician to make participation in this study inadvisable); 8) significant cognitive deficit resulting in an inability to understand or comply with instructions; 9) surgery planned in the next year; 10) Intention to move away from Ottawa region in the next year; 11) an inability to communicate in English or French; or 12) an unwillingness to sign informed consent. The study coordinator assessed the inclusion/exclusion criteria of all potential participants by telephone. At the first visit, written informed consent was obtained if the patient met all inclusion criteria.

### Intervention

Participants were randomly assigned to one of the three KT intervention groups using central randomization [37] and computer generated numbers [38]: 1) Walking and Behavioural intervention (WB) (18 males, 57 females) which included the SCAWP with a behavioural intervention and an educational pamphlet on the benefits of walking for OA; 2) Walking intervention (W) (24 males, 57 females) wherein participants only received the SCAWP intervention and the educational pamphlet; 3) Self-directed control (C) (32 males, 52 females) wherein participants only received the educational pamphlet. All 3 groups were provided with pedometers and log books to be completed to measure physical performance (walking in minutes) and additional PA aside from the walking sessions (Table 1). The KT strategies were implemented over a 12-month duration and participants were assessed for additional 6 months (15 and 18-month follow-up assessments).

**Table 1:** Summary of the KT strategies used

**KT strategies in each group**		
WB or WalkingClub+ (supervised walking program + behavioural approach at the community-based Walking Club)	W or WalkingClub (supervised walking program only at the community-based Walking Club)	C or Self-directed Home-based or community based different than W and WB (unsupervised/self-directed Walking program only)
EBP walking program (Ottawa Panel, 2005/2012) refs: [33-35]	EBP walking program (Ottawa Panel, 2005/2012) refs: [33-35]	Walking program (general info from pamphlet on walking and OA)
Walking Club + effect (supervised program, team, daily monitored vital measures: BP, HR, #steps,)	Walking Club effect (supervised program, team, daily monitored vital measures: BP, HR, #steps,)	N/A
Pedometer as measurement tool, but becomes a KT strategy	Pedometer as measurement tool, but becomes a KT strategy	Pedometer as measurement tool, but becomes a KT strategy
Log book including 7-day PAR as measurement tool, but becomes a KT strategy	Log book including 7-day PAR as measurement tool, but becomes a KT strategy	Log book including 7-day PAR as measurement tool, but becomes a KT strategy
$ compensation each walking session and at evaluation session	$ compensation each walking session and at evaluation session	$ compensation each logbook fulfilled and at evaluation session
Study affiliation/participation (feel committed)	Study affiliation/participation (feel committed)	Study affiliation/participation (feel committed)
Behavioural intervention (Baycrest PACE-ex: patient education + goal settings; PA counselling, telephone support)	N/A	N/A

Following the successful randomization process, wherein no statistical difference was found between the three groups (with the exception of the “physical role” category of the SF-36: part II) (Tables 2, 3, 4, 5), research staff and evaluators were blinded to the treatment allocation. An independent evaluator was blinded to assess outcome measures. Due to the nature of the physical intervention, it was not practical to blind the study participants and PA specialists supervising the aerobic walking program [39].

**Table 2:** Subject demographics and baseline characteristics

**Characteristic**	**Walking (n=79)**	**Walking and Behavioural (n=69)**	**Self-directed Control (n=74)**	**Total**
**Mean age (SD), yrs**	63.9 (10.3)	63.9 (8.2)	62.3(6.8)	63.4 (8.6)
*Missing Data*	*0*	*0*	*0*	*0*
**Men/women, (%)**	24 (30.4)/55 (69.9)	18 (26.1)/51 (73.9)	27 (36.5)/47 (63.5)	69 (31.1)/153 (68.9)
*Missing Data*	*0*	*0*	*0*	*0*
**Affected knee, n (%)**				
Right	33 (41.8)	31 (44.9)	25 (33.8)	89 (40.1)
Left	31(39.2)	23 (33.3)	28 (37.8)	82 (36.9)
Both side	15 (19.0)	15 (28.4)	21 (28.4)	51 (23.0)
*Missing Data*	*0*	*0*	*0*	*0*
**Mean duration of OA (SD), yrs**	9.54 (8.09)	11.3 (9.7)	10.0 (9.9)	10.3 (9.26)
*Missing Data*	*0*	*0*	*0*	*0*
**Mean weight (SD), kg**	80.7 (18.5)	83.1 (15.4)	83.0(15.8)	82.2 (16.6)
*Missing Data*	*0*	*1*	*0*	*0*
**Mean BMI (SD), kg/m²**	29.4 (5.4)	30.3 (5.6)	29.9(5.3)	29.8 (5.4)
*Missing Data*	*0*	*2*	*4*	*6*
**Walking aid, n (%)**				
Yes	10 (12.7)	10 (14.5)	9 (12.2)	29 (13.1)
No	69 (87.3)	58 (84.1)	64 (86.5)	191 (86.0)
*Missing Data*	*0*	*1*	*1*	*2*
**Racial background, n (%)**				
White	69 (87.3)	60 (87.0)	68 (91.9)	197 (88.7)
Black	1 (1.3)	3 (4.3)	1 (1.4)	5 (2.3)
Hispanic	2 (2.5)	2 (2.9)	4 (5.4)	8 (3.6)
Asian or Pacific Islander	5 (6.3)	4 (5.8)	1 (1.4)	10 (4.5)
American Indian or Alaskan native	1 (1.3)	0 (0)	0 (0)	1 (0.5)
Other	1 (1.3)	0 (0)	0 (0)	1 (0.5)
*Missing Data*	*0*	*0*	*0*	*0*
**Marital status, n (%)**				
Married	46 (58.2)	36 (52.2)	44 (59.5)	126 (56.8)
Separated	2 (2.5)	1 (1.4)	1 (1.4)	4 (1.8)
Divorced	9 (11.4)	17 (24.6)	8 (10.8)	34 (15.3)
Widowed	17 (21.5)	11 (15.9)	9 (12.2)	37 (16.7)
Never Married	5 (6.3)	4 (5.8)	12 (16.2)	21 (9.5)
*Missing Data*	*0*	*0*	*0*	*0*
**Level of education, n (%)**				
Less than 7 yrs of school	2 (2.5)	1 (1.4)	1 (1.4)	4 (1.8)
Grades 7 through 9	5 (6.3)	0 (0)	0 (0)	5 (2.3)
Grades 10 through 11	7 (8.9)	4 (5.8)	5 (6.8)	16 (7.2)
High school graduate	13 (16.5)	16 (23.2)	8 (10.8)	37 (16.7)
1 to 4 yrs of college	13 (16.5)	9 (13.0)	22 (29.7)	44 (19.8)
College graduate	25 (31.6)	21 (30.4)	19 (25.7)	65 (29.3)
Professional or Graduate school	14 (17.7)	18 (26.1)	19 (25.7)	51 (23.0)
*Missing Data*	*0*	*0*	*0*	*0*

Yrs: years, OA: osteoarthritis, kg: kilograms, m: meters, %: validity percent.

**Table 3:** Subject’s Medication at Baseline

**Medication**	**Walking (W) only (n=79)**	**Walking and Behavioural (WB) (n=69)**	**Self-directed (C) (n=74)**
Oral hypoglycaemic agents			
Total	2 (3)	5 (7)	9 (12)
Biguanide Class	2 (3)	4 (6)	9 (12)
Sulfonylureas Class	0 (0)	0 (0)	2 (3)
Thiazolidinedione	0 (0)	2 (3)	2 (3)
Antihypertensive Agents			
Total	22 (28)	15 (22)	15 (22)
Calcium channel blockers	6(8)	0(0)	7(9)
Antiotensin-converting enzyme	3 (4)	7 (10)	7 (9)
Angiotensin II receptor blockers	6 (8)	6 (9)	3 (4)
Beta-blocker	4 (5)	3 (4)	4 (5)
Alpha-blocker	3 (4)	0 (0)	0 (0)
Diuretic	5 (6)	8 (12)	5 (7)
Antiarrhythmic	1 (1)	0 (0)	0 (0)
Anti-anginal	1 (1)	0 (0)	0 (0)
Anti-platelet agent	1 (1)	1 (1)	1 (1)
Anticoagulant	0 (0)	2 (3)	0 (0)
Phosphodiesterase type 5 inhibitors	0 (0)	1 (1)	0 (0)
Statins	8 (10)	12 (17)	10 (14)
Hormone			
Total	11 (14)	10 (14)	11 (15)
Thyroid	5 (6)	5 (7)	8 (11)
Insulin	0 (0)	3 (4)	1 (1)
Corticosteroid	4 (5)	2 (3)	3 (4)
Progesterone	1 (1)	0 (0)	1 (1)
Androgen	1 (1)	0 (0)	0 (0)
Oestrogen	1 (1)	0 (0)	0 (0)
Antithyroid	0 (0)	1 (1)	0(0)
SERMs (Selective estrogen receptor modifiers)	1 (1)	0 (0)	0 (0)
Beta 2-adrenergic receptor agonist (bronchodilator)	2(3)	1 (1)	3 (4)
NSAIDs (Non-steroid anti-inflammatory drugs)	26 (33)	21 (30)	18 (24)
COX-2 selective inhibitor	3 (4)	4 (6)	1 (1)
NSAIDS			
Betahistine	0 (0)	1 (1)	0 (0)
DMARDs (Disease modifying antirheumatic drugs)	0 (0)	1 (1)	0 (0)
Histamine antagonist	0 (0)	2 (3)	0 (0)
Bisphosphonate	6 (8)	4 (6)	4 (5)
Antibiotics	2 (3)	1 (1)	4 (5)
Analgesics	1 (1)	1 (1)	3 (4)
Opiates	0 (0)	0 (0)	2 (3)
Hypnotic	4 (5)	3 (4)	4 (5)
Antidepressant	7 (9)	5 (7)	7 (9)
Psychostimulant	0 (0)	1 (1)	0 (0)
Antipsychotic	0 (0)	1 (1)	0 (0)
Anti-manic	0 (0)	1 (1)	0 (0)
Anti-convulsant	1 (1)	1 (1)	0 (0)
Protein pump inhibitor (PPI)	9 (11)	5 (6)	6 (8)
Enzyme inhibitor	0 (0)	1 (1)	2 (3)
Muscle relaxant	0 (0)	1 (1)	1 (1)
Antispasmodic	0 (0)	1 (1)	0 (0)
Anti-cholinergic	1 (1)	1 (1)	0 (0)
Antineoplastic	2 (3)	1 (1)	0 (0)
Antimetabolite	1 (1)	1 (1)	1 (1)
Antifolate	0 (0)	1 (1)	1 (1)
Latanoprost	0 (0)	1 (1)	0 (0)
Viscosupplementation	0 (0)	1 (1)	0 (0)
Xanthine	1 (1)	0 (0)	0 (0)
Antifungals	0 (0)	0 (0)	1 (1)
Antimalarial	0 (0)	1 (1)	1 (1)
Nitrate	0 (0)	0 (0)	2 (3)
Laxative	0 (0)	0 (0)	2 (3)
Supplements	22 (28)	22 (28)	18 (24)
Homeopathic Medication	1 (1)	1 (1)	1 (1)
None	11 (14)	10 (13)	9 (12)
Not Specific	21 (27)	21 (27)	20 (27)
Missing Data			
Total	4 (5)	8 (10)	17 (23)
No Evaluation*	2 (3)	4 (5)	7 (9)
No File**	2 (3)	4 (6)	10 (14)

This table presents a list of medications which participants declared to be taking at baseline. Data is presented as n (%).

**Table 4:** Summary of adherence based on attendance marked in trainers’ manuals and individual walkers’ logbooks

	**Walking N Mean±SD**	**Walking & Behavioural N Mean±SD**	**Self-directed (C) N Mean±SD**	**Walking vs. Self-directed *t*-test P value**	**Walking & Behavioural Vs. Self-directed *t*-test P value**	**Walking & Behavioural Vs. Walking *t*-test P value**
0-3 months	79	69	73	*(0.043)	*(0.012)	(0.514)
	0.770±0.299	0.802±0.290	0.652±0.403			
3-6 months	79	69	73	(0.242)	(0.159)	(0.774)
	0.617±0.410	0.636±0.390	0.535±0.459			
6-9 months	79	69	73	(0.421)	(0.937)	(0.363)
	0.471±0.418	0.534±0.425	0.528±0.463			
9-12 months	79	69	73	(0.549)	(0.551)	(0.989)
	0.446±0.441	0.445±0.433	0.490±0.462			
Total Adherence	79	69	73	(0.690)	(0.413)	(0.619)
	0.576±0.346	0.604±0.342	0.551±0.420			

**Table 5:** Summary of self-efficacy, measured with Stanford questionnaire on chronic disease for three study arms (continued)

	**Baseline**			**12 months**			**18 months**		
	**W**	**WB**	**C**	**W**	**WB**	**C**	**W**	**WB**	**C**
	**N**	**N**	**N**	**N**	**N**	**N**	**N**	**N**	**N**
Variables	Mean±SD	Mean±SD	Mean±SD	Mean±SD	Mean±SD	Mean±SD	Mean±SD	Mean±SD	Mean±SD
				W.Vs. C(P)	W.Vs. C(P)	W.Vs. W(P)	W.Vs. C(P)	W.Vs. C(P)	W.Vs. W(P)
Coping	79	69	73	44	44	40	44	41	36
With	1.069±794	1.126±0.775	1.013±0.946	0.864±0.771	1.239±0.730	1.192±0.835	1.064±0.952	1.388±0.856	1.342±1.127
Symptoms				(0.091)	(0.858)	(0.057)	(0.190)	(0.793)	(0.286)
Confidence	79	69	73	44	44	39	43	41	36
About	7.406±1.719	7.827±1.488	7.858±1.512	7.826±1.551	7.682±1.785	8.064±1.575	7.690±1.920	7.546±1.848	8.015±1.476
Doing Things				(0.433)	(0.297)	(0.060)	(0.235)	(0.422)	*(0.041)

(Higher is better) W: Walking only group; WB: Walking and Behavioural Group; C: Self-directed group (unsupervised/self-directed); N: number of subjects in each comparative group; SD: standard deviation; vs: versus; data is presented as mean (standard deviation); p: p-value (statistical significance); * Statistically significant.

#### Supervised walking programs (WB & W groups)

The SCAWPs took place at two walking club sites in Ottawa, Ontario, Canada and one in Gatineau, Québec, Canada. Participants took part in three weekly walking sessions over a 12-month period. Every walking session began with a 10-minute warm-up, consisting of light aerobic exercises, before engaging in the 45-minute aerobic walking phase. The walking sessions ended with a 10-minute cool-down consisting of light aerobic exercises and stretching. The target intensity of the walking phase was approximately 50% to 70% of the subjects’ pre-determined maximum heart rate as recommended in The Ottawa Panel guidelines [33-35]. The SCAWP was divided into two stages: 1) A “progressive aerobic phase” wherein the duration and heart rate intensity progressively increased over time and 2) a “maintenance aerobic phase” wherein the duration and heart rate intensity remained constant for the remainder of the walking program (Additional file 2). The selected dosage, frequency, intensity, and progression of the walking interventions were based on existing protocols proven effective for OA, described in several RCTs involving individuals with OA [33-35] (Additional file 2). To avoid contamination between the two walking interventions, participants in the W group were instructed to walk in the mornings, while the WB group walked in the afternoons at the main walking site.

In order to meet the prescribed target heart rate, heart rate monitors were provided to each participant prior to his/her walking session. As a safety precaution, participants who experienced prolonged pain lasting more than two hours within 24 hours after a walking session were asked to temporarily reduce their level of exercise until he/she felt comfortable enough to resume the appropriate duration, intensity and frequency of the walking program. Following randomization, the PA specialist met each subject in the walking groups to explain the SCAWP and its progression (Additional file 2) and was present for a minimum of three weekly scheduled sessions over one year to supervise the subjects. The PA specialist was responsible for monitoring and recording attendance, blood pressure, heart rate (during and after the walking session), time duration, and number of steps (pedometer) at each walking session. Monetary compensation was given to each participant for attending each walking session.

#### Behavioural intervention (WB group)

The behavioural intervention was part of the multifaceted KT intervention (Table 1) and was implemented using the adapted *Program for Arthritis Control through Education and Exercise* (PACEex) program [40]. The behavioural intervention was integrated into the PACEex program and consisted of the following components: (1) short- and long-term goal setting during PACEex classes; (2) an educational component, delivered by a trained instructor, involving consisting of instructional sessions about the benefits of PA, specifically walking; (3) monthly face-to-face counselling wherein participants received moral support/encouragement to adherence with PA. Potential barriers to program adherence were identified and self-management strategies were reviewed to overcome those barriers; (4) goal setting and telephone counselling were provided as an additional source of social support until the end of the supervised phase. As with the face-to-face counselling, barriers were identified and strategies were shared in an effort to promote the long-term maintenance of PA. In summary, the behavioural intervention consisted of twenty 2-hour group sessions discussing short-term goal setting and education of arthritis-related topics over a duration of 20 consecutive weeks. Individual long-term goal setting was discussed at the beginning of the PACEex program and was followed by monthly face-to-face meetings throughout the first 6 months of the program. The last six months of the 12-month supervised phase consisted of participants receiving counselling via telephone discussing long-term goals and barriers/facilitators to adhere to the walking program.

#### The self-directed control (C group)

Participants were invited to consult an educational pamphlet on walking and OA. One introductory session was provided to the participant wherein they were provided an educational pamphlet describing the benefits of walking for OA, a pedometer to monitor PA, and a logbook to record activity level and adherence while partaking in the self-directed walking program. Participants in this group received monetary compensation for the completion his/her log books. The self-directed (C) group did not have any contact with participants in the two other groups, avoiding potential contamination.

### Data collection

Participants were assessed by an independent evaluator at baseline and at each 3 month interval (months 3, 6, 9, 12) during the intervention period. Participants were then assessed at 3 and 6 months post-intervention during the follow-up period (15 and 18-months). Participants were asked to complete a collection of validated questionnaires as well as perform physical tests at each assessment.

## Results

### Knowledge (CPG) uptake outcome measures (KT outcomes)

#### Adherence and behaviour change

Similar to other studies [29,41,42], participant adherence was assessed as the number of attended walking sessions divided by the number of prescribed sessions (3 sessions per week). Among the W and WB groups, the number of attended walking sessions throughout the 12-month duration was recorded by the exercise therapist on site as well as through the use of participants’ completed logbooks which provided information on the amount of performed PA during the 12-month intervention period. The C group’s adherence was only assessed by the completed log books. Behaviour change was based on the concept of self-efficacy and was measured using the Stanford scale. In regards to the research question, behaviour change results were only reported at 12 and 18-months.

#### Analysis

The analysis was conducted on an intention-to-treat basis. Descriptive statistics including mean, standard deviation and frequencies were used to summarize the study groups at baseline. A repeated measure mixed model was used to assess the change in adherence from baseline to end of treatment (12 months) among three study groups. Behavioural change outcomes were assessed from baseline to of end treatment (12 months) and at 6-month follow-up post-intervention (18 months). The model included variables such as intervention group, study month, and an interaction term between intervention group and study month. Missing data was assumed to be missing at random (MAR) in order to include incomplete data. Pairwise differences comparing each group to one another (W vs. C, W vs. WB, WB vs. C) were assessed. The repeated measure mixed model was used to compare the change of adherence from baseline to the 18- month follow-up. Statistical analyses were performed using SAS (version 9.2, SAS Institute Inc., Cary, North Carolina), and statistical significance was defined as p<0.05.

## Results

**Figure 2:**
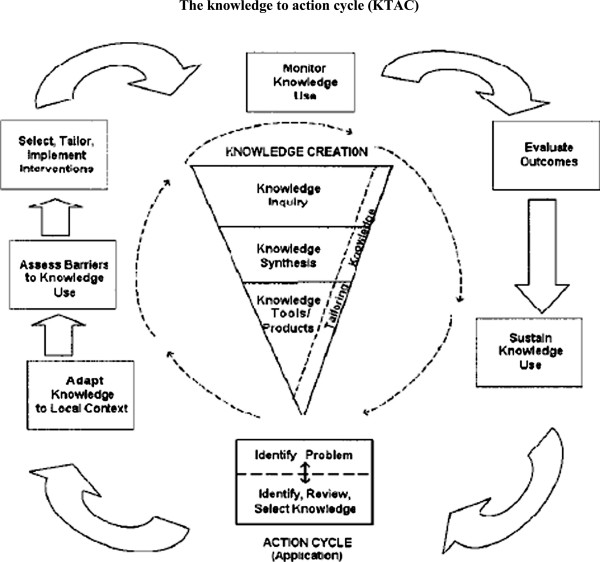
**Study flow diagram.** This figure provides a flow diagram of data collection from baseline to 18-months follow-up.

Between January 2007 and December 2008, 629 study participants were assessed for eligibility. Figure 2 exhibits the flow of participants from recruitment to follow-up. The most common reasons for exclusion were: 1) time commitment (26.5%); 2) first diagnosed with OA by physician or X-Ray but later contradicted by MRI test result (18.1%); 3) loss of interest (14.2%); 4) severe OA of the weight bearing joint of LE (11.8%); 5) no medical clearance after VO2 max test (5.1%); 6) unable to contact (4.9%); 7) too active (2,9%); 8) knee replacement (2.7%); 9) age related (2.5%); 10) travelling too much (1.9%); 11) living too far (1.2%). Follow-up for the last recruited participant was completed in May 2010.

The groups were similar in age, sex, ethnicity, OA duration and medication use (Table 2 &3). Two hundred and twenty two subjects were included in the study. Women made up 153 of the 222 subjects. The mean age (±S.D.) was 63.4 (±8.6) years, the mean disease duration (±S.D.) was 10.3 (±9.3) years, the highest level of completed education was “College graduate” with 65 participants (±29.3) and “Professional or graduate school” with 51 participants (±23.0). There was no statistical difference between the three comparative groups at baseline for all the demographic variables.

### Blinding

The effectiveness of blinding the evaluator to treatment allocation was assessed through a questionnaire where she was asked to identify which group each participant belonged to. The blinding rate of the evaluator was high (98%).

### Adherence and attrition

From baseline to 12 months, the adherence rates were expressed as percentages of adherence among walkers who did not drop out and were reported as means (+/−standard deviation) for each time period between groups (Table 4). Statistically significant results for short-term adherence rates favoured participants in the WB group (80.2%) compared to the self-directed (C) group (65.2%) (p<0.012) after 3 months. For long term adherence (6 to 12 months), the WB group demonstrated superior adherence rates throughout each period compared to the other two groups, but results did not reach statistical significance. No statistical significance (p> 0.05) was observed for the total adherence rates between WB vs. W, W vs. C, and WB vs. C.

During the 12-month intervention period, the dropout rates were 43.1% for the W group, 40.6% for the WB group and 49.3% for the self-directed (C) group. From baseline to 18 months, the dropout rates were 44.3% for the W group; 40.6% for the WB group and 52.1% for the self-directed (C) group (Figure 2 and Additional files 3 &4). The dropout rates were lower for the WB group at 12-months (36.2%) and 18-months (39.1%) compared to the W group (40.5% and 44.3%) and the self-directed (C) group (40.5% and 51.3%). The W and self-directed (C) groups had the highest dropout rates (40.5%) at 12 months while the self-directed (C) group had the highest at 18 months (52.1%).

### Behaviour change

After the 12 month intervention phase, no differences were found between the 3 comparative groups for any of the items on the Stanford scale (Table 5). Moreover, variables related to coping with symptoms and confidence were higher among the WB group compared to the other two groups.

At 18-months, statistically significant differences in scores between the WB and W groups revealed that the W group demonstrated an improved mean for the “confidence about doing things” variable (p=0.041). The self-directed group (C) demonstrated the highest mean “confidence about doing things” score 8.015± 1.476 (p=0.040) as it increased by 0.157 (p=0.048) when compared to baseline. The self-directed group (C) demonstrated the highest mean score 8.015± 1.476 (p=0.040).

## Discussion

### The Impact of the multifaceted KT interventions on knowledge (CPG) uptake

#### Adherence and attrition

The additional behavioural approach of the multifaceted KT intervention (WB group) improved adherence to the SCAWP when compared to the other two groups over a short-term period. In addition, the WB group demonstrated reduced dropout rates compared to the W and self-directed (C) groups. Over a long-term period, the three groups were equivalent in improving behaviour change while adopting the Ottawa Panel recommendations on walking [33-35]. Adherence rates were higher among the WB group for each 3-month period compared to the other two groups. Improved adherence rates over the short-term period can be explained by the fact the PACEex program used in the behavioural intervention lasted for 20 consecutive weeks at the beginning of the study. The PACEex program [40] used in this study, allowed our participants to set general goals, which may have motivated participants to adhere to the walking program. Similar to a previous study [43], another reason may be that participants in the W and WB group lost motivation after the supervised phase, no longer having regular professional support from the PA specialist.

The drop-out rate results are comparable to other long-term studies which have used an aerobic PA intervention with a behavioural intervention component [38,44-54]. Higher adherence rates were demonstrated at 2 to 4 months (85% - 90%) and at 10 to 18 months (50% - 90%). An initial 6-month center-based exercise program enhanced retention over short and the long-term periods while promoting short-term adherence and energy expenditure [55]. The moderate exercise intervention retained more subjects, but had little influence on adherence over a long-term period [56]. Home-based PA programs, such as self-directed walking, can achieve improved adherence rates compared to facility-based programs over a long period [55].

### Behaviour change

There were no statistically significant results among the three groups at 12 months. These results concur with a similar study [53] and are normal in the context of a KT study which implemented an already proven effective walking program [33-35]. However, at 18-months, there were statistically significant results which favoured the self-directed group (C). One reason explaining why participants in the self-directed group (C) had higher confidence and self-efficacy scores may be due to the fact that since these individuals were not supervised, they may have been more independent, self-controlled, self-confident, and self-motivated. Participants in this group may have also had the opportunity, over the 12-month period, to develop confidence and self-efficacy skills compared to the two supervised groups during the unsupervised follow-up phase.

The variable “confidence about doing things” decreased after 12 months for the WB group. Given the high amount of PA demonstrated by the WB group at 12 months, we were surprised with this result. On the other hand, at 18 months, statistically significant results favoured the W group compared to WB group for the variables “confidence about doing things”. Again, these results about confidence were unexpected given that the WB group performed higher amounts of PA.

Exercise therapists, pedometers, logbooks and social support from walking club members (Table 1) assisted participants who had difficulties with activity adherence and maintenance [57]. Regular professional contact was recognised as the significant motivational factor for adherence to the supported walking program during the RCT [43]. Social support has been reported to be more successful in engaging participants in PA programs compared to programs which only provided written educational material [58,59]. The combination of using pedometers and goal-setting has been recognised as an effective KT tool for increasing PA [31,60-62]. However, despite the short-term effectiveness of pedometers and goal-setting, the success of these tools diminished over time. Other KT strategies are recommended to facilitate sustainability [62].

### Limitations

Barriers related to the implementation of SCAWP were identified and addressed to improve adherence for older individuals [57]. Acceptability (preferences, tolerance and accessibility) should be considered to promote PA adherence and to overcome associated barriers [63]. The most common barriers identified by participants with OA are perceptions about illness and recovery, transportation difficulties, family commitments and inconvenient timing [64]. Time commitment is also recognised as a significant barrier [55]. Additional barriers such as weather conditions including snow storms, freezing rain and humidity, a 3-month bus strike, and health problems of participants’ partners may have contributed to study adherence.

Adherence is influenced by participant’s preference of intervention [65-70]. Unfortunately, with this study being a RCT, taking participants’ preference into consideration was not possible due to the randomization procedure. This being said, randomisation may have contributed to higher dropout rates. The purpose of this KT study was to implement a scientifically proven effective walking program for OA [33-35].

Short term research suggests that self-management interventions [16,18], telephone counselling [22,46,71-73], peer support [13,74-76] and PA education [18,22,27,41,77] are effective behavioural strategies for improving self-efficacy. The long-term multifaceted behavioural strategy used in this study did not concur with the previous studies, as participants in the self-directed group (C group) were provided information and education on the benefits of PA.

Methods of measurement for adherence for the W and WB groups were different than the self-directed (C) group. Observed attendance was recorded on site for each walking session for the W and WB groups by exercise therapists while adherence could only be measured in the self-directed group (C) using completed logbooks. The self-reporting of PA, which is commonly overstated, may have been subject to potential information bias.

Selection bias may have occurred as our study population included individuals with mild to moderate OA, with a confirmed X-ray report. In addition, some participants were retired while some were still employed. Since participants were provided monetary compensation for their participation in the study, one may argue the generalizability of the results.

### Implications

This KT RCT aimed to identify the best multifaceted strategy to implement a proven effective walking program for OA of the knee among older individuals. The epidemic public health problem of OA can be managed through the implementation of a proven effective walking program in existing community-based walking clubs as well as at home with minimal support. More studies are needed to improve the long-term walking adherence or knowledge uptake on SCAWP among participants with OA. Particular attention should focus on patients’ characteristics and preferences.

## Conclusions

This study partially supported our initial hypothesis regarding the impact of a multifaceted KT intervention as the combined walking and behavioural approach had greater benefits over the comparative groups resulting in improved long-term adherence to PA only over a short period (3-months). The WB group demonstrated to be the best KT strategy for reducing dropouts compared to the W group and self-directed (C) group. All three KT strategies were equivalent over the long-term period (up to 18 months) for improving behavioural change. However, the self-directed walking program (C group) was the least expensive to implement over a long-period (18 months). This RCT was a long-term adherence study as well as a KT study which addressed questions of clinical and scientific importance aimed at improving the understanding of effective KT strategies to promote the adoption and maintenance of a community-based walking program for OA.

## Abbreviations

OA: Osteoarthritis; QoL: Quality of Life; SCAWP: Supervised Community-based Aerobic Walking Program; CPG: Clinical Practice Guideline; KT: Knowledge Translation; KTAC: Knowledge-To-Action Cycle; EBCPG: Evidence-Based Clinical Practice Guidelines; CONSORT: Consolidated Standards of Reporting Trials; WB (group): Walking and Behavioural intervention; W (group): Walking intervention; C (group): Self-directed control intervention; RCT: Randomized Controlled Trial; PA: Physical Activity; PACE-ex: Program for Arthritis Control through Education and Exercise; AIMS2: Arthritis Impact Measurement Scale 2; SF-36: Short-Form 36 Health Survey; WOMAC: Western Ontario and McMaster Universities Osteoarthritis Index; MET: Multiples of basal metabolic rate; MAR: Missing At Random; CIHR: Canadian Institutes of Health Research.

## Competing interests

The authors declare that they have no competing interests.

## Authors’ contributions

LB is a Full Professor of Rehabilitation, an epidemiologist and the principal investigator of this study and primary author of this manuscript. GAW the co-principal investigator of this study, is senior biostatistician and director, Cardiovascular Research Methods Centre at the University of Ottawa Heart Institute, and is a leading expert in the design and analysis of clinical trials. He provided assistance with the methodology and statistical analysis of the study. GPK is a Full Professor of Physiology at the University of Ottawa, and director of the university’s Human Performance and Environmental Medicine Research Laboratory and Professional Fitness and Lifestyle Consultant Certification Training program. He assisted with the methodology of the study. RR is a senior researcher at the University of Ottawa Heart Institute and provided experience in applying innovative behavioural approaches aimed at increasing PA in healthy and chronic diseased populations. AM is a health economist and assisted with the economic evaluation concept in the original proposal. PT is a rheumatologist, an epidemiologist and chief of the Cochrane Musculoskeletal Group. He has experience in conducting RCTs and meta-analyses. He provided assistance with OA outcome measures in the study. MH & CM developed the PACEex program and was in charge of training the PA specialist. GDA was the research coordinator of this study and assisted with the writing of this manuscript. LC is a biostatistician and performed the analyses of this study. All authors read and approved the final manuscript.

## Pre-publication history

The pre-publication history for this paper can be accessed here:

http://www.biomedcentral.com/1471-2458/12/871/prepub

## Supplementary Material

Additional file 1 **The knowledge –to-action framework and KT interventions.** This table demonstrates how each step of the KTAC framework is applied to the RCT. Click here for file

Additional file 2 **Post-Randomization Walking Interventions.** The supervised phase of the walking intervention lasted 52 weeks following randomization. The unsupervised (follow-up) phase of the walking intervention lasted 26 additional weeks. For Intervention (one year): 52 weeks × 3 sessions =156 sessions attended; Follow-up (six month): 26 weeks x 3 sessions = 78 sessions attended. N.B. This structured and supervised walking program is based on Ottawa Panel Grade A recommendations [33-35] and was implemented to groups W and WB (Implementation groups). Individuals with OA in the self-directed group (C) received a pamphlet on OA and walking (which recommends regular walking in a unsupervised/self-directed way) (Dissemination group) [25]. Click here for file

Additional file 3 **Reasons of dropouts.** This table demonstrates the reasons as to why participants decided to withdraw from the study. Click here for file

Additional file 4 **Dropout rates and corresponding retention rates at 12-and 18-month time periods.** This table demonstrates the drop-out rates and retention rates at end of intervention 12-months and follow-up at 18-months. Click here for file

## References

[B1] Losina E Walensky RP Reichmann WM Holt HL Gerlovin H Solomon DH Jordan JM Hunter DJ Suter LG Weinstein AM Paltiel AD Katz JN Impact of obesity and knee osteoarthritis on mortality in older Americans Ann Intern Med 2011 154 4 217 226 21320937 10.1059/0003-4819-154-4-201102150-00001 PMC3260464

[B2] Jordan JM Helmick CG Renner JB Luta G Dragomir AD Woodard J Fang F Schwartz TA Abbate LM Callahan LF Kalsbeek WD Hochberg MC Prevalence of knee symptoms and radiographic and symptomatic knee osteoarthritis in African Americans and Caucasians: the Johnson Country Osteoarthritis Project J Rheumatol 2007 34 1 172 180 17216685

[B3] Grotle M Hagen KB Natvig B Dahl FA Kvien TK Prevalence and burden of osteoarthritis: results from a population survey in Norway J Rheumatol 2008 35 4 677 684 18278832

[B4] Vetter NJ Effect of an aging population on service use Reviews in Clinical Gerontology 2005 15 55 62 10.1017/S0959259805001693

[B5] Denton FT Spencer BG Chronic health conditions: Changing prevalence in an aging population and some implications for the delivery of health care services Can J Aging 2010 29 1 11 21 10.1017/S0714980809990390 20202262

[B6] Wright EA Katz JN Cisternas MG Kessler CL Wagenseller A Losina E Impact of knee osteoarthritis on health care resource utilization in a US population-based national sample Medical Care 2010 48 9 785 791 10.1097/MLR.0b013e3181e419b1 20706165 PMC3258446

[B7] Liu-Ambrose TY Ashe MC Marra C The physical and chronic conditions research team. Independent and inverse association of healthcare utilisation with physical activity in older adults with multiple chronic conditions Br J Sports Med 2010 44 14 1024 1028 10.1136/bjsm.2008.046458 18487254

[B8] Graham ID Logan J Harrison MB Straus SE Tetroe J Caswell W Robinson N Lost in knowledge translation: time for a map? J Contin Educ Health Prof 2006 26 1 13 24 10.1002/chp.47 16557505

[B9] Straus SE Tetroe J Graham ID Knowledge translation in health care: moving from evidence to practice 2009 Wiley-Blackwell/BMJ, Chichester 318

[B10] Kanouse DE Kallich JD Kahan JP Dissemination of effectiveness and outcomes research Health Policy 1995 34 3 167 192 10.1016/0168-8510(95)00760-1 10153899

[B11] Estabrroks CA Translating research into practice: Implications for organizations and administrators Can J Nurs Res 2003 35 3 53 68 14603570

[B12] Grimshaw J Freemantle N Wallace S Russell I Hurwitz B Watt I Long A Sheldon T Developing and implementing clinical practice guidelines Qual Health Care 1995 4 1 55 64 10.1136/qshc.4.1.55 10142039 PMC1055269

[B13] Sharpe PA Community-based physical activity intervention Arthritis Rheum 2003 49 3 455 462 10.1002/art.11054 12794804

[B14] Hootman JM Macera CA Ham SA Helmick CG Sniezek JE Physical activity levels among the general US adult population and in adults with and without arthritis Arthritis Rheum 2003 49 1 129 135 10.1002/art.10911 12579604

[B15] Boutaugh ML Arthritis Foundation community-based physical activity programs: Effectiveness and implementation issues Arthritis Rheumatism (Arthritis Care and Research) 2003 49 3 463 470 10.1002/art.11050 12794805

[B16] Minor M Stenström CH Klepper SE Hurley M Ettinger WH Work Group Recommendations: 2002 Exercise and Physical Activity Conference –Session V: evidence of benefit of exercise and physical activity in arthritis, St. Louis, Missouri. Arthritis Rheumatism 2003 49 3 453 454 10.1002/art.11125 12794803

[B17] Chang R Roubenoff R Mayer J Work Group Recommendations: 2002 Exercise and Physical Activity Conference – Session IV: exercise in the presence of rheumatic disease, St. Louis, Missouri Arthritis Rheumatism (Arthritis Care and Research) 2003 49 2 280 10.1002/art.11011 12687524

[B18] Brady TJ Sniezek JE Implementing the national arthritis action plan: New population-based approaches to increasing physical activity among people with arthritis Arthritis & Rheumatism (Arthritis Care and Research) 2003 49 3 471 476 10.1002/art.11052 12794806

[B19] Zhang W Moskowitz RW Nuki G Abramson S Altman RD Arden N Bierma-Zeinstra S Brandt KD Croft P Doherty M Dougados M Hochberg M Hunter DJ Kwoh K Lohmander LS Tugwell P OARSI recommendations for the management of hip and knee osteoarthritis, Part II: OARSI evidence-based, expert consensus guidelines Osteoarthritis Cartilage 2008 16 2 137 162 10.1016/j.joca.2007.12.013 18279766

[B20] Devos-Comby L Cronan T Roesch SC Do exercise and self-management interventions benefit patients with osteoarthritis of the knee? A metaanalytic review J Rheumatol 2006 33 4 744 756 16583478

[B21] Laforest S Nour K Gignac M Gauvin L Parisien M Poirier M-C Short-term effects of self-management intervention on hralth status of housebound older adults with arthritis J Appl Gerontol 2008 27 5 539 567 10.1177/0733464808319712

[B22] Hurley MV Muscle dysfunction and effective rehabilitation of knee osteoarthritis outcomes: What we need to find out Arthritis Rheum 2003 49 3 444 452 10.1002/art.11053 12794802

[B23] Grimshaw JM Shirran L Thomas RE Mowatt G Fraser C Bero L Grilli R Harvey EL Oxman AD O’Brien MA Changing provider behaviour: an overview of systematic reviews of interventions Medical Care 2001 39 Supplement 2 II-2 II-45 11583120

[B24] Bero L Grilli R Grimshaw JM Harvey E Oxman AD Thomson MA Closing the gap between research and practice: an overview of systematic reviews of interventions to promote implementation of research findings by health care professionals Br Med J 1998 317 465 468 10.1136/bmj.317.7156.465 9703533 PMC1113716

[B25] Van der Wees PJ Jamtvedt G Rebbeck T de Bie RA Dekker J Hendriks EJ Multifaceted strategies may increase implementation of physiotherapy clinical guidelines: a systematic review Aust J Physiother 2008 54 4 233 241 10.1016/S0004-9514(08)70002-3 19025503

[B26] Brosseau L Egan M Wells GA Tugwell P Dubouloz CJ Casimiro L Welch V McEwan J Ottawa Panel evidence-based clinical practice guidelines for patient education programs in the treatment of osteoarthritis Health Educ J 2010 70 318 358

[B27] Kirk S Diet and weight management Nurs Stand 2003 17 49 47 53 12953376 10.7748/ns2003.08.17.49.47.c3442

[B28] McKay HG King D Eakin EG Seeley JR Glasgow RE The diabetes network internet-based physical activity intervention: a randomized pilot study Diabetes Care 2001 24 8 1328 1334 10.2337/diacare.24.8.1328 11473065

[B29] Talbot LA Gaines JM Huynh TN Metter EJ A home-based pedometer-driven walking program to increase physical activity in older adults with osteoarthritis of the knee: A preliminary study Journal of American Geriatric Society 2003 51 3 387 392 10.1046/j.1532-5415.2003.51113.x 12588583

[B30] Zoellner J Powers A Avis-Williams A Ndirangu M Strickland E StricklYadrick K Compliance and Acceptability of Maintaining a 6-Month Pedometer Diary in a Rural, African American Community-Based Walking Intervention J Phys Act Health 2009 6 4 475 482 19842462 10.1123/jpah.6.4.475

[B31] Tudor-Locke CE Myers AM Challenger and opportunities for measuring physical activity in sedentary adults Sports Med 2001 31 2 91 100 10.2165/00007256-200131020-00002 11227981

[B32] Schulz KF Atman DG Moher D Consort 2010 Statement: updated guidelines for reporting parallel group randomised trials Journal of Pharmacology and pharmacotherapeutics 2010 1 2 100 107 10.4103/0976-500X.72352 21350618 PMC3043330

[B33] Brosseau L Wells G Tugwell P Egan M Dubouloz CJ Casimiro L Robinson V Pelland L McGowan J Lamb M Ottawa Panel evidence-based clinical practice guidelines for therapeutic exercises and manual therapy in the treatment of osteoarthritis Phys Ther 2005 85 9 907 971 16117601

[B34] Brosseau L Wells GA Tugwell P Egan M Dubouloz CJ Casimiro L Bugnariu N Welch V De Angelis G Francoeur L Milne S Loew L McEwan J Messier SP Doucet E Kenny GP Prud’homme D Lineker S Bell M Poitras S Li JX Finestone HM Laferrière L Haines-Wangda A Russell-Doreleyers M Lambert K Marshall AD Cartizzone M Teav A Ottawa Panel evidence-based clinical practice guidelines for the management of osteoarthritis in adults who are obese and overweight Phys Ther 2011 91(6 843 861 21493746 10.2522/ptj.20100104

[B35] Loew L Brosseau L Wells G Tugwell P Egan M Dubouloz CJ Casimiro L Welch V McEwan J Ottawa Panel evidence-based clinical practice guidelines for walking programs in the treatment of osteoarthritis Arch Phys Med Rehabil 2012 93 7 1269 1285 10.1016/j.apmr.2012.01.024 22421624

[B36] Scott J Huskisson EC Graphic representation of pain Pain 1976 2 2 175 184 10.1016/0304-3959(76)90113-5 1026900

[B37] Van Tulder MW Assendett WJ Koes BW Bouter LM Method guidelines for systematic reviews in the Cochrane Collaboration Back Review Group for spinal disorders: Operationalization of Van Tulder’s quality assessment form Spine 1997 22 20 2323 2330 10.1097/00007632-199710150-00001 9355211

[B38] Jadad AR Moore RA Carroll D Jenkinson C Reynolds DJ Gavaghan DJ McQuay HJ Assessing the Quality of Reports of Randomized Clinical Trials: Is Blinding Necessary? Control Clin Trials 1996 17 1 1 12 10.1016/0197-2456(95)00134-4 8721797

[B39] Deyo RA Walsh NE Schoenfeld LS Ramamurthy S Can trials of physical treatments be blinded? The example of transcutaneous electrical nerve stimulation for chronic pain American Journal of Physiology & Medicine Rehabilitation 1990 69 6 10 10.1097/00002060-199002000-00003 2137345

[B40] Mendelson AD McCullough C Chan A Integrating Self-Management and Exercise for People Living with Arthritis Health Educ Res 2010 26 1 167 177 21123844 10.1093/her/cyq077

[B41] Meyers A Compliance with exercise therapy in treating seniors with knee osteoarthritis Clinical Journal of Sports Medicine 1998 8 148 10.1097/00042752-199804000-00022 9641447

[B42] Hughes SL Seymour RB Campbell R Pollak N Huber G Sharma L Impact of the fit and strong intervention on older adults with osteoarthritits Gerontologist 2004 44 2 217 228 10.1093/geront/44.2.217 15075418

[B43] Coghill N Cooper AR Motivators and de-motivators for adherence to a program of sustained walking Prev Med 2009 49 24 27 10.1016/j.ypmed.2009.04.017 19426757

[B44] Kovar PA Allegrante JP Mackenzie CR Peterson MG Gutin B Charlson ME Supervised fitness walking in patients with osteoarthritis of the knee. A randomized, controlled trial Ann Intern Med 1992 116 7 529 534 1543305 10.7326/0003-4819-116-7-529

[B45] Messier SP Thompson CD Ettinger MH Effects of long-term aerobic or weight training regimens on gait in an older, osteoarthritic population J Appl Biomech 1997 13 202 225

[B46] Messier SP Loeser RF Miller GD Morgan TM Rejeski WJ Sevick MA Ettinger WH Pahor M Williamson JD Exercise and dietary weight loss in overweight and obese older adults with knee osteoarthritis. The arthritis, diet and activity promotion trial Arthritis Rheum 2004 50 5 1501 1510 10.1002/art.20256 15146420

[B47] Peloquin L Bravo G Gauthier P Lacombe G Billiard JS Effects of a cross-training exercise program in persons with osteoarthritis of the knee. A randomized controlled trial J Clin Rheumatol 1999 5 126 136 10.1097/00124743-199906000-00004 19078371

[B48] Penninx BW Messier SP Rejeski WJ Williamson JD Dibari M Cavazzini C Applegate WB Pahor M Physical exercise and the prevention of disability in activities of daily living in older persons with osteoarthritis Arch Intern Med 2001 161 19 2309 2316 10.1001/archinte.161.19.2309 11606146

[B49] Peterson MG Kovar-Toledano PA Otis JC Allegrante JP Mackenzie CR Gutin B Kroll MA Effect of a Walking Program on Gait Characteristics in Patients with Osteoarthritis Arthritis Care Res 1993 6 1 11 16 10.1002/art.1790060104 8443252

[B50] Rejeski WJ Brawley LR Ettinger W Morgan T Thompson C Compliance to exercise therapy in older participants with knee osteoarthritis: Implications for treating disability Medicine & Science in Sport & Exercise 1997 29 8 977 985 10.1097/00005768-199708000-00001 9268953

[B51] Sullivan T Allegrante JP Peterson MG Kovar PA Mackenzie CR One-year follow up of patients with osteoarthritis of the knee who participated in a program of supervised fitness walking and supportive patient education Arthritis Care Res 1998 11 4 228 233 10.1002/art.1790110403 9791321

[B52] Sharma L Cahue S Song J Hayes K Pai Y-C Dunlop D Physical Functioning Over Three Years in Knee Osteoarthritis Arthritis Rheum 2003 48 12 3359 3370 10.1002/art.11420 14673987

[B53] Minor MA Hewett JE Webel RR Anderson SK Kay DR Efficacy of physical conditioning exercise in rheumatoid arthritis and osteoarthritis Arthritis Rheum 1989 32 11 1396 1405 10.1002/anr.1780321108 2818656

[B54] Minor MA Brown JD Exercise maintenance of persons with arthritis after participation in a class experience Health Educ Q 1993 20 1 83 95 10.1177/109019819302000108 8444627

[B55] Ashworth NL Chad KE Harrison EL Reeder BA Marshall SC Home versus center based physical activity programs in older adults Cochrane Database Syst Rev 2005 1 CD004017 15674925 10.1002/14651858.CD004017.pub2 PMC6464851

[B56] Cox KL Burke V Gorely TJ Beilin LJ Puddey IB Controlled Comparison of Retention and Adherence in Home- vs Center-Initiated Exercise Interventions in Women Ages 40–65 Years: The S.W.E.A.T. Study (Sedentary Women Exercise Adherence Trial) Prev Med 2003 36 1 17 29 10.1006/pmed.2002.1134 12473421

[B57] Poitras S Rossignol M Avouac J Avouac B Cedrasch C Nordin M Rousseaux C Rozenberg S Savarieau B Thoumie P Valat JP Vignon E Hillinquin P Management recommendations for knee osteoarthritis: How usable are they? Joint Bone Spine 2010 77 5 458 465 10.1016/j.jbspin.2010.08.001 20851659

[B58] Kirk AF Higgins LA Hughes AR Fisher BM Mutrie N Hillis S Maclntyre PD A randomized, controlled trial to study: the effect of exercise consultation on the promotion of physical activity in people with type 2 diabetes: a pilot study Diabet Med 2001 18 11 877 882 10.1046/j.0742-3071.2001.00570.x 11703431

[B59] Wankel LM Decision making and social support strategies for increasing physical involvement Journal Cardiac 1984 4 124 135

[B60] Merom D Rissel C Phongsavan P Smith BJ Van Kemenade C Brown WJ Bauman AE Promoting walking with pedometers in the community: the step-by-step trial Am J Prev Med 2007 32 4 290 297 10.1016/j.amepre.2006.12.007 17303369

[B61] Bravata DM Smith-Spangler C Sundaram V Gienger AL Lin N Lewis R Stave CD Olkin I Sirard JR Using pedometers to increase physical activity and improve health A systematic review. JAMA 2007 298 19 2296 2304 10.1001/jama.298.19.2296 18029834

[B62] Baker G Mutrie N Lowry R Using pedometers as motivational tools: are goals set in steps more effective than goals set in minutes for increasing walking? Int. J. Health Promotion 2008 46 21 26

[B63] Vignon E Valat JP Rossignol M Avouac B Rozenberg S Thoumie P Anouac J Nordin M Hilliquin P Osteoarthritis of the knee and hip and activity: a systematic international review and synthesis (OASIS) Joint Bone Spine 2006 73 4 442 455 10.1016/j.jbspin.2006.03.001 16777458

[B64] Beswick AD Rees K West RR Taylor FC Burke M Griebsch I Improving uptake and adherence in cardiac rehabilitation: literature review J Adv Nurs 2005 49 5 538 555 10.1111/j.1365-2648.2004.03327.x 15713186

[B65] Burke SM Carron AV Shapcott KM Cohesion in exercise groups: an overview International Review of Sport and Exercise Psychology 2008 1 2 107 123 10.1080/17509840802227065

[B66] Torgerson DJ Klaber-Moffett J Russell IT Patient preferences in randomised trials: threat or opportunity? J Health Serv Res Policy 1996 1 4 194 197 10180870 10.1177/135581969600100403

[B67] Torgerson DJ Sibbald B Understanding controlled trials. What is a patient preferencetrial? BMJ 1998 316 7128 360 10.1136/bmj.316.7128.360 9487173 PMC2665528

[B68] Brewin CR Bradley C Patient preferences and randomised clinical trials Br Med J 1989 299 6694 313 315 10.1136/bmj.299.6694.313 2504416 PMC1837157

[B69] McPherson K Chalmers I Incorporating patient preferences into clinical trials BMJ 1998 317 7150 78 10.1136/bmj.317.7150.78 9651286 PMC1113468

[B70] Dunn G The challenge of patient choice and non-adherence to treatment in randomized controlled trials of counseling or psychotherapy Understand Stat 2002 1 19 29 10.1207/S15328031US0101_03

[B71] Evcik D Sonel B Effectiveness of a home-based exercise therapy and walking program on osteoarthritis of the Knee Rheumatol Int 2002 22 3 103 106 10.1007/s00296-002-0198-7 12111084

[B72] Ettinger WH Burns R Messier SP Applegate W Rejeski WJ Morgan T Shumaker S Berry MJ O’Toole M Monu J Craven T A randomized trial comparing aerobic exercise and resistance exercise with a health education program in older adults with knee osteoarthritis: The Fitness Arthritis and Seniors Trial (FAST) J Am Med Assoc 1997 277 1 25 31 10.1001/jama.1997.03540250033028 8980206

[B73] Green BB MaAfee T Hindmarsk M Madsen L Coplow M Buist D Effectiveness of telephone support in increasing physical activity level in primary care patients American Journal of Preventive Medecine 2002 22 3 177 183 10.1016/S0749-3797(01)00428-7 11897462

[B74] Westby MD A health professional’s guide to exercise prescription for people with arthritis: a review of aerobic fitness activities Arthritis Rheumatism (Arthritis Care Res) 2001 45 6 501 511 10.1002/1529-0131(200112)45:6<501::AID-ART375>3.0.CO;2-Y 11762684

[B75] Sherwood NE Jeffrey RW The behavioral determinants of exercise: implications for physical activity interventions Annu Rev Nutr 2000 20 21 44 10.1146/annurev.nutr.20.1.21 10940325

[B76] Marcus BH Sallis JF Lean AS Determinants of physical activity behavior and implications for interventions Physical activity and cardiovascular health: a national concensus champaign 1997 192, Illinois 192 201

[B77] Van der Bij AK Laurant MG Wensing M Effectiveness of physical activity interventions for older adults: A review Am J Prev Med 2002 22 2 120 133 10.1016/S0749-3797(01)00413-5 11818183

