# Supplementary material for: The implementation of a community-based aerobic walking program for mild to moderate knee osteoarthritis (OA): a knowledge translation (KT) randomized controlled trial (RCT): Part I: The Uptake of the Ottawa Panel clinical practice guidelines (CPGs)
Source: BMC Public Health. 2012 Oct 13;12:871. doi: 10.1186/1471-2458-12-871 (PMC3491047; doi:10.1186/1471-2458-12-871)
Supplement: Additional file 1 — The knowledge –to-action framework and KT interventions. This table demonstrates how each step of the KTAC framework is applied to the RCT. [file 1471-2458-12-871-S1.pdf]

Phase	Description
Knowledge Creation	In this study, knowledge was previously created through the use of Ottawa Panel CPGs (2005; 2011; In Press, 2012) and the People Getting a Grip on Arthritis Program (www.arthritis.ca/peoplegettingagrip) (Brosseau ,2011)
Action Cycle	
Identify the knowledge-to-action gaps	What is the most effective strategy to improve participant compliance to proven effective structured community-based aerobic walking program and improve behaviour change after 12 months among individuals with OA
Adapt knowledge to local context	Implementing a scientifically proven effective structured community-based aerobic walking program to local walking clubs in Ottawa, Ontario, Canada, and Gatineau, Quebec, Canada
Assess barriers to knowledge use	Strengths and barriers were assessed in a previous pilot study and focus group investigating preferred methods of physical activity. In addition, walking progression and recommendations for OA of the knee were assessed in a previous systematic review (Ottawa Panel, 2005)
Select, Tailor, Implement Interventions	The KT interventions were multifaceted consisting either an educational pamphlet (C group), a structured community-based aerobic walking program (W group), or the combination of patient education, a structured community-based aerobic walking program and behavioural techniques such as patient education, goal setting, health counselling, telephone support (WB group).
Monitor knowledge use	Knowledge use was monitored through the supervision of the structured community-based aerobic walking program and its progression by a physical activity specialist at the walking club. The physical activity specialist was present for a minimum of three weekly sessions (Monday, Wednesday and Friday) over 12 months to supervise the participants in the W and WB groups and to record vital signs and attendance.
Evaluate outcomes	Outcomes included knowledge (guidelines) uptake measured by compliance and behavioural changes (self-efficacy and confidence) to the structured community-based aerobic walking program based on Ottawa Panel CPGs (2005; 2011; In Press, 2012) and behavioural change (self-efficacy and confidence) in adopting the SCAWP. Measurement sessions were scheduled every three months over the course of the 18-month project (See part II for more details on outcome evaluation for quality of life and clinical outcomes)
Sustained Knowledge Use	Volunteers at the existing walking clubs took over during the follow-up phase between 12 and 18 months and will hopefully implement the same long-term program beyond the study.
