# Supplementary material for: The implementation of a community-based aerobic walking program for mild to moderate knee osteoarthritis (OA): a knowledge translation (KT) randomized controlled trial (RCT): Part I: The Uptake of the Ottawa Panel clinical practice guidelines (CPGs)
Source: BMC Public Health. 2012 Oct 13;12:871. doi: 10.1186/1471-2458-12-871 (PMC3491047; doi:10.1186/1471-2458-12-871)
Supplement: Additional file 2 — Post-Randomization Walking Interventions. The supervised phase of the walking intervention lasted 52 weeks following randomization. The unsupervised (follow-up) phase of the walking intervention lasted 26 additional weeks. For Intervention (one year): 52 weeks × 3 sessions =156 sessions attended; Follow-up (six month): 26 weeks x 3 sessions = 78 sessions attended. N.B. This structured and supervised walking program is based on Ottawa Panel Grade A recommendations [33-35] and was implemented to groups W and WB (Implementation groups). Individuals with OA in the self-directed group (C) received a pamphlet on OA and walking (which recommends regular walking in a unsupervised/self-directed way) (Dissemination group) [25]. [file 1471-2458-12-871-S2.pdf]

		INDIVIDUAL AEROBIC WALKING TRAINING		
**Week No.**	**Phase**	**Duration (min/day)**	**Intensity (% HRmax)**	**Frequency (days/wk)**
1-4	Progression	25	50	3
5-8	Progression	30	55	3
9-10	Progression	35	55	3
11-12	Progression	40	60	3
13-16	Progression	45	60	3
17-19	Progression	45	65	3
20-26	Progression	45	70	3
27-52	Maintenance	45	70	3
