# Supplementary material for: The implementation of a community-based aerobic walking program for mild to moderate knee osteoarthritis (OA): a knowledge translation (KT) randomized controlled trial (RCT): Part I: The Uptake of the Ottawa Panel clinical practice guidelines (CPGs)
Source: BMC Public Health. 2012 Oct 13;12:871. doi: 10.1186/1471-2458-12-871 (PMC3491047; doi:10.1186/1471-2458-12-871)
Supplement: Additional file 3 — Reasons of dropouts. This table demonstrates the reasons as to why participants decided to withdraw from the study. [file 1471-2458-12-871-S3.pdf]

Reasons	W	WB	C	Total
Unable to contact after admission	8	2	10	20
No motivation	0	2	1	3
No MD clearance after 2nd VO2max test	0	3	3	6
Time commitment	5	3	5	13
No OA when based on X-Rays or MRI	1	2	2	5
Health problems	7	7	7	21
Transportation	1	2	0	3
Personal/family reasons	7	1	2	10
Knee surgery	2	0	3	5
Injury	3	2	1	6
Did not like structure of program	0	1	1	2
No specific reason	0	1	3	3
Moved away	1	2	0	3
**Total**	**35**	**28**	**38**	**101**
