# Supplementary material for: The implementation of a community-based aerobic walking program for mild to moderate knee osteoarthritis (OA): a knowledge translation (KT) randomized controlled trial (RCT): Part I: The Uptake of the Ottawa Panel clinical practice guidelines (CPGs)
Source: BMC Public Health. 2012 Oct 13;12:871. doi: 10.1186/1471-2458-12-871 (PMC3491047; doi:10.1186/1471-2458-12-871)
Supplement: Additional file 4 — Dropout rates and corresponding retention rates at 12-and 18-month time periods. This table demonstrates the drop-out rates and retention rates at end of intervention 12-months and follow-up at 18-months. [file 1471-2458-12-871-S4.pdf]

Time periods	W group	WB group	Self-directed group (C)
**Supervised phase at 12 months**			
Dropout rate	34/79 (43.1%)	28/69 (40.6%)	36/73 (49.3%)
Retention rate	45/79 (56.9%)	41/69 (59.4%)	37/73 (50.7%)
**Follow-up phase at 18 months**			
Dropout rate	35/79 (44.3%)	28/69 (40.6%)	38/73 (52.1%)
Retention rate	44/79 (55.7%)	41/69 (59.4%)	35/73 (47.9%)
